# Early Abrogation of Gelatinase Activity Extends the Time Window for tPA Thrombolysis after Embolic Focal Cerebral Ischemia in Mice

**DOI:** 10.1523/ENEURO.0391-17.2018

**Published:** 2018-06-27

**Authors:** Shanyan Chen, Zhenzhou Chen, Jiankun Cui, Myah L. McCrary, Hailong Song, Shahriar Mobashery, Mayland Chang, Zezong Gu

**Affiliations:** 1 Department of Pathology and Anatomical Sciences, University of Missouri at Columbia, Columbia, MO 65212; 2 Interdisciplinary Neuroscience Program, University of Missouri at Columbia, Columbia, MO 65212; 3 Harry S. Truman Memorial Veterans' Hospital Research Service, Columbia, MO 65201; 4 Department of Chemistry and Biochemistry, University of Notre Dame, Notre Dame, IN 46556

**Keywords:** cerebral ischemia, endothelial transcytosis, matrix, MMP-9, SB-3CT, tissue plasminogen activator

## Abstract

Acute ischemic stroke (AIS) is caused by clotting in the cerebral arteries, leading to brain oxygen deprivation and cerebral infarction. Recombinant human tissue plasminogen activator (tPA) is currently the only Food and Drug Administration-approved drug for ischemic stroke. However, tPA has to be administered within 4.5 h from the disease onset and delayed treatment of tPA can increase the risk of neurovascular impairment, including neuronal cell death, blood-brain barrier (BBB) disruption, and hemorrhagic transformation. A key contributing factor for tPA-induced neurovascular impairment is activation of matrix metalloproteinase-9 (MMP-9). We used a clinically-relevant mouse embolic model of focal-cerebral ischemia by insertion of a single embolus of blood clot to block the right middle cerebral artery. We showed that administration of the potent and highly selective gelatinase inhibitor SB-3CT extends the time window for administration of tPA, attenuating infarct volume, mitigating BBB disruption, and antagonizing the increase in cerebral hemorrhage induced by tPA treatment. We demonstrated that SB-3CT attenuates tPA-induced expression of vascular MMP-9, prevents gelatinase-mediated cleavage of extracellular laminin, rescues endothelial cells, and reduces caveolae-mediated transcytosis of endothelial cells. These results suggest that abrogation of MMP-9 activity mitigates the detrimental effects of tPA treatment, thus the combination treatment holds great promise for extending the therapeutic window for tPA thrombolysis, which opens the opportunity for clinical recourse to a greater number of patients.

## Significance Statement

Endovascular thrombectomy benefited patients with disproportionately severe clinical deficit relative to infarct volume and provides a surgical option for treatment of ischemic stroke. Tissue plasminogen activator (tPA) is the only Food and Drug Administration-approved clot-dissolving drug for treatment of ischemic stroke. However, its beneficial effect is seen only within 4.5 h from the onset of stroke, with better outcomes observed the sooner treatment is given. Major risk is matrix metalloproteinase-9 (MMP-9)-mediated neurovascular impairment. We document that early administration of the pharmacological agent SB-3CT reduces MMP-9 levels and mitigates the side effects associated with tPA. Furthermore, it extends the time window for treatment with tPA after stroke holding great potential for both disease intervention and benefiting patients who might not otherwise be candidates for treatment with tPA.

## Introduction

Stroke affects ∼795,000 individuals in the United States and kills 130,000 Americans every year (Mozaffarian et al., 2015). Acute ischemic stroke (AIS) is the most common form of stroke, accounting for 87% of strokes (Mozaffarian et al., 2015), and is primarily caused by thrombosis or embolism blocking the cerebral arteries. Cerebral ischemia initiates a cascade of pathologic events, including vasogenic edema, disruption of the blood-brain barrier (BBB), intracerebral hemorrhage (ICH), and neuronal cell death (Moskowitz et al., 2010).

Tissue plasminogen activator (tPA) is the only Food and Drug Administration-approved drug for treatment of AIS. It is a serine protease, which generates plasmin from its inactive precursor plasminogen. Plasmin accelerates the clearance of fibrin deposits from the blood clot, thus restores cerebral blood flow (CBF) and oxygen supply in the ischemic regions. However, tPA has to be administered within 4.5 h from the onset of stroke to be effective (Hacke et al., 2008) and is contraindicated in patients with hemorrhagic stroke. As a result, <5% of stroke patients are eligible for tPA therapy (Donnan et al., 2011). Furthermore, tPA itself has been demonstrated to also promote neuronal degeneration after cerebral ischemia (Wang et al., 1998). Delayed treatment with tPA is accompanied by degradation of extracellular matrix (ECM) components and increased BBB permeability (Su et al., 2008), as well as enhanced hemorrhagic transformation (Lapchak et al., 2000).

The neurotoxic side effects of tPA result from the tPA-promoted activation of brain matrix metalloproteinase-9 (MMP-9) after focal cerebral ischemia (Tsuji et al., 2005). Thus, combination of tPA with MMP inhibition has been proposed as a treatment strategy for AIS. MMPs, a family of 23 zinc-dependent endopeptidases in humans, regulate many components of the ECM during tissue homeostasis and remodeling (Yong, 2005). Evidence shows that the expression of zymogenic proMMP-9 is elevated and that laminin, a substrate of MMPs, is decreased in humans after stroke, with active MMP-9 seen only in patients treated with tPA (Horstmann et al., 2003). We and others have shown that aberrant MMP-9 proteolytic activity is associated with increased permeability of the BBB, which results in brain edema and hemorrhage, and directly contributes to neuronal apoptosis and brain damage after AIS (Gasche et al., 1999b; Lapchak et al., 2000; Gu et al., 2002).

SB-3CT is the first selective mechanism-based slow-binding gelatinase inhibitor (Brown et al., 2000). The root of the selectivity of SB-3CT lies in a reaction catalyzed by the target gelatinases that results in ring-opening of the thiirane moiety to the corresponding thiolate, which coordinates with zinc as a tight-binding picomolar inhibitor, for which the reversal of inhibition occurs very slowly (Forbes et al., 2009). As tPA activates MMP-9 and exacerbates hemorrhagic transformation after cerebral ischemia (Sumii and Lo, 2002; Lo, 2008), we hypothesized that the use of SB-3CT in combination with tPA could mitigate neurotoxicity and extend the time window for treatment by tPA in a physiologically relevant model of AIS in mice. We document herein that this is indeed the case.

## Materials and Methods

### Mouse model of embolic focal cerebral ischemia

All animal experiments were performed in a blinded manner and approved by the Author University Animal Care and Usage Committee. The mouse embolic model of focal cerebral ischemia was described previously (Cui et al., 2012). Briefly, adult male C57BL/6J mice (The Jackson Laboratory, 7–11 weeks of age and 18–27 g body weight) were used in this study. A single 10-mm length of autologous clot was inserted from the external carotid artery into the circle of Willis to occlude the origin of the MCA. A laser Doppler flowmeter was used to measure regional CBF (rCBF). The initial measurement of rCBF was assigned 100%, and subsequent readings were expressed relative to this value. Monitoring of rCBF showed mice with successful cerebral ischemia to <25% of preischemic baselines. Animals were not included in the subsequent study if rCBF measured at 2 h after occlusion increased to 4-fold of the rCBF of sustained ischemia. The gelatinase selective inhibitor SB-3CT was prepared as a 12.5 mg/ml solution formulated in vehicle [25% DMSO/65% polyethylene glycol 200 (PEG-200)/10% H_2_O]. Mice were intraperitoneally injected with 2 μl/gram body weight of this solution (equivalent to 25 mg/kg) at 2 h postischemia, followed by a second dose of SB-3CT at 4 h and tPA (prepared in saline at 2.5 mg/kg) given intravenously at 4 h after embolic ischemia. A second group of mice was given SB-3CT intraperitoneally at 2, 4, and 6 h postischemia, followed by tPA intravenously at 6 h after ischemia. For administration of tPA, 10% of total volume was injected in the first 1 min, and the remaining tPA was infused within 30 min using a pump. All mice were decapitated and the brains were harvested at 24 h after cerebral ischemia.

### Measurement of infarct percentage

Mouse brains were processed using the stereology technique as previously described (Garden et al., 2002; Helton et al., 2005; Cui et al., 2012; Hadass et al., 2013). Briefly, coronal sections were serially cut at 40-µm thickness with a vibrotome (VT1200S, Leica Microsystems, Inc.). Stained sections were analyzed using an Olympus BX-41 upright pathology microscope (Olympus America Inc.). Infarct percentage was quantified using ImageJ software. To minimize errors associated with edema, infarct area was indirectly calculated. Briefly, nonlesioned brain areas in the ischemic and contralateral hemispheres were measured; the percentage of infarct percentage relative to the area measured in the contralateral hemisphere: (areas of contralateral hemisphere, areas of nonlesioned ischemic hemisphere)/areas of contralateral hemisphere *100% (Cui et al., 1999, 2012; Gu et al., 2005; Zhao et al., 2006).

### Measurement of IgG extravasation

For IgG extravasation detection, brain sections were blocked with 2% normal goat serum for 2 h at room temperature, then incubated with biotin-conjugated goat anti-mouse IgG antibody (115-065-003, Jackson ImmunoResearch) at 1:1000 dilution for 2 h at room temperature. Sections were then incubated with ABC Elite (Avidin/Biotin) Systems (Reagent A&B from PK-6101, Vector Laboratories) and visualized with 3, 3’-diaminobenzidine tetrahydrochloride Liquid Substrate System (D3939, Sigma Chemical Co; Ruth and Feinerman, 1988; Chen et al., 1999; ElAli et al., 2011). Stained sections were analyzed using an Olympus BX-41 upright pathology microscope and quantified using ImageJ software. IgG extravasation is calculated as percentage of IgG-positive areas: (area of contralateral hemisphere – IgG-negative area in ischemic hemisphere)/area of contralateral hemisphere *100%. IgG-positive areas were quantified using ImageJ software.

### Quantification of intracerebral hemorrhagic volume

Spontaneous ICHs were quantified in cresyl violet-stained serial sections (Garden et al., 2002; Cui et al., 2012). ICH was observed and identified using a 4× objective lens of Olympus BX-41 upright pathology microscope. Additional validation and capturing images was conducted by using a 10× or 20× objective lens. Each hemorrhage was individually measured using the ImageJ software. Hemorrhage volumes were stereologically calculated by integrating the section interval thickness (200 µm).

### Immunofluorescence labeling

After transcardiac perfusion with phosphate-buffered saline and 4% paraformaldehyde (PFA), mouse brains were removed and placed in 4% PFA for 1 h and submerged in 30% sucrose overnight at 4°C. Coronal cryosections (20 µm thick) were cut from optimal cutting temperature-embedded mouse brains. For immunofluorescence labeling, brain sections were immunostained with the following primary antibodies: the endothelial marker CD31 (1:50, 550274; BD Biosciences); the ECM basement membrane component laminin (1:200, L9393; Sigma Chemical Co), MMP-9 (1:100, MAB13415, EMD Millipore, Merck KGaA), and caveolin-1 (1:1000, ab18199, Abcam). Sections were visualized with fluorophore conjugated secondary antibodies (1:300, goat anti-rat IgG-Alexa Fluor 594, A11007; goat anti-mouse IgG-Alexa Fluor 488, A11001; and goat anti-rabbit IgG-Alexa Fluor 488, A110034; ThermoFisher). Nuclear DNA dye Hoechst 33342 was used for counterstain (Gu et al., 2005). The ischemic penumbra was identified with areas of condensed nucleus changing to round, pale-stained nucleus gradually. Fluorescence images were taken with a Leica DMI 6000B microscope (Leica Microsystem). Image capture, 3D deconvolution and analysis were analyzed with LAS AF analysis tools. 3D deconvolution was used to enhance sharpness and contrast of some fluorescent images.

### Measurement of vascular density

Images with colocalization of laminin and CD31 immunoreactivity in the penumbra region were used for quantification of vascular density. The densities of laminin and CD31-positive vessels were quantified separately using ImageJ software with Vessel Analysis plugins, following a protocol from http://imagej.net/Vessel_Analysis, as previous described (Morin et al., 2015). The vascular density was calculated with Vessel Analysis plugins according to the following equation:

vascular density = vessel area/total are × 100%

### Statistics

Prism 5 software (GraphPad Software) was used for data analysis. Unpaired one-tailed Student’s *t* test was applied to compare differences between two groups based on predictions formulated with prior data (Gu et al., 2005; Cui et al., 2012). Data are presented as means ± SEM. A value of *p < 0.05 was considered statistically significant.

## Results

### SB-3CT in combination with tPA protects against brain damage after embolic cerebral ischemia

**Figure 1.**
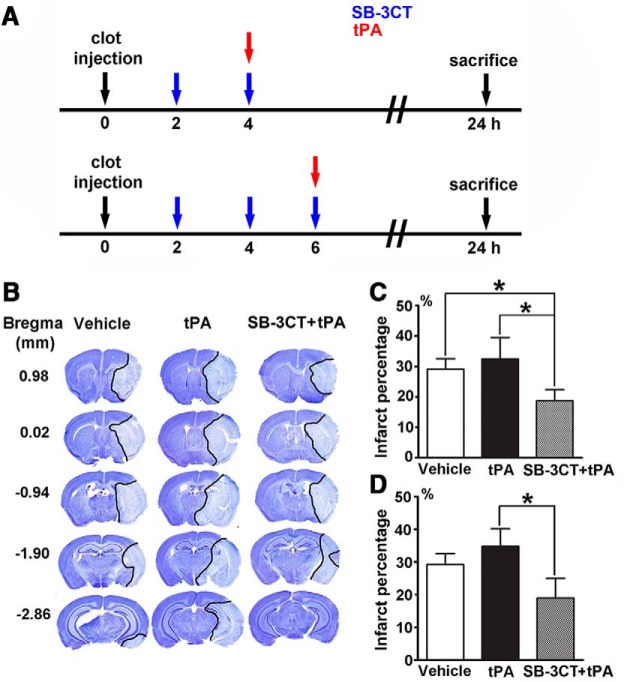
Effect of SB-3CT on brain damage in tPA-treated mice after embolic ischemia. ***A***, Experimental design for embolic MCAO and SB-3CT and tPA administration. SB-3CT was given intraperitoneally at 25 mg/kg; tPA was administered intravenously at 2.5 mg/kg. Vehicle controls (25% DMSO/65% PEG-200/10% water intraperitoneally and saline intravenously) were included. ***B***, Representative cresyl violet-stained images at different distances from bregma showing infarct areas (light blue regions marked by black lines). Infarct percentage for tPA given at 4 h (***C***) or 6 h (***D***) postischemia; *n* = 10 mice for vehicle, *n* = 6 for tPA, and *n* = 7 for SB-3CT + tPA groups; *p < 0.05 by one-tailed Student’s *t* test. Data are expressed as mean ± SEM.

We evaluated the effect of SB-3CT in combination with tPA in mice after embolus-induced middle cerebral artery occlusion (MCAO), closely manifesting the pathologic conditions of fibrin-rich clot derived ischemic stroke (Cui et al., 2012). Mice were treated with SB-3CT or vehicle at 2, 4, and 6 h postischemia, followed by treatment with tPA or saline at 4 or 6 h (Fig. 1*A*). SB-3CT treatment was started at 2 h postischemia since MMP-9 expression increases in the brain as early as 2 h after AIS (Gasche et al., 1999a). A second dose of SB-3CT was administered 2 h later to maintain brain concentrations above the *K*_i_ value (Cui et al., 2012). Brains from all groups were collected at 24 h after cerebral ischemia. The mean infarct percentage after accounting for edema, as indicated by staining of brain sections with cresyl violet (Fig. 1*B*), was 29.2 ± 3.4% in the vehicle group, 32.5 ± 7.0% in the tPA group, and 18.8 ± 3.7% in the SB-3CT + tPA at 4 h (Fig. 1*C*). Combined treatment with SB-3CT + tPA at 4 h resulted in a significant reduction of infarct percentage compared to the vehicle-treated mice (*p* < 0.05) and to the tPA-treated mice. Mice treated with SB-3CT + tPA at 6 h postischemia, had also significantly lower (∼45%) infarct percentage (18.9 ± 6.1%) than mice treated only with tPA (34.7 ± 5.4%, *p* < 0.05; Fig. 1*D*). The infarct percentage was not significantly different (*p* = 0.40) between the mice treated with tPA at 4 h (32.5 ± 7.0%) and 6 h (34.7 ± 5.4%) postischemia. These findings indicate that SB-3CT in combination with tPA protects against brain damage and counteracts the tPA-mediated neurotoxicity.

### SB-3CT in combination with tPA protects against BBB leakage

**Figure 2.**
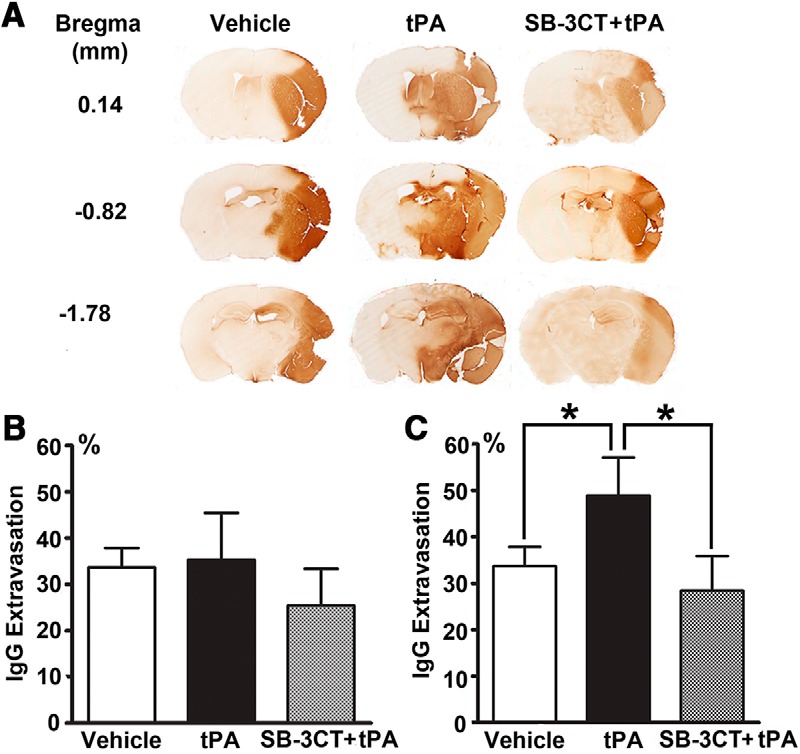
Effect of SB-3CT on BBB leakage in tPA-treated mice after embolic ischemia. ***A***, BBB leakage as ascertained by IgG extravasation. Representative brain sections from the same mice described in Figure 1 were stained with goat anti-mouse IgG. IgG extravasation for tPA given at 4 h (***B***) or 6 h (***C***) postischemia; *p < 0.05 by one-tailed Student’s *t* test.

BBB disruption after embolic cerebral ischemia was evaluated by endogenous plasma protein leakage using IgG extravasation as an indicator. Endogenous IgG is present at high levels in plasma and absent in brain parenchyma when the BBB is intact. Thus, IgG immunoreactivity in the brain parenchyma can be used to monitor protein extravasation and BBB disruption (ElAli et al., 2011). IgG extravasation was not detected in the contralateral hemisphere, but it markedly increased in the ischemic hemisphere (Fig. 2*A*). No significant differences in IgG extravasation in the ischemic hemispheres were observed between the vehicle, tPA, and SB-3CT + tPA-treated mice at 4 h postischemia (Fig. 2*B*), while tPA treatment at 6 h postischemia significantly increased the IgG extravasation by >40% compared to the vehicle-treated mice (Fig. 2*C*). SB-3CT in combination with tPA potently protected from BBB leakage, with a significant reduction in IgG extravasation to 28.5 ± 7.4% of the contralateral hemisphere compared to 48.9 ± 8.2% in the tPA-treated mice (Fig. 2*C*). These findings indicate that tPA causes dramatic BBB damage when given at 6 h, but not at 4 h postischemia. Our results are consistent with those from the European Cooperative Acute Stroke Study, which indicate that the time window for tPA treatment can be extended between 3.0 and 4.5 h (Hacke et al., 2008). Importantly, our study shows that the time window for tPA treatment can be further extended to 6 h by combination treatment with SB-3CT as it counteracts the tPA-induced BBB disruption.

### SB-3CT in combination with tPA attenuates cerebral hemorrhage after embolic cerebral ischemia

**Figure 3.**
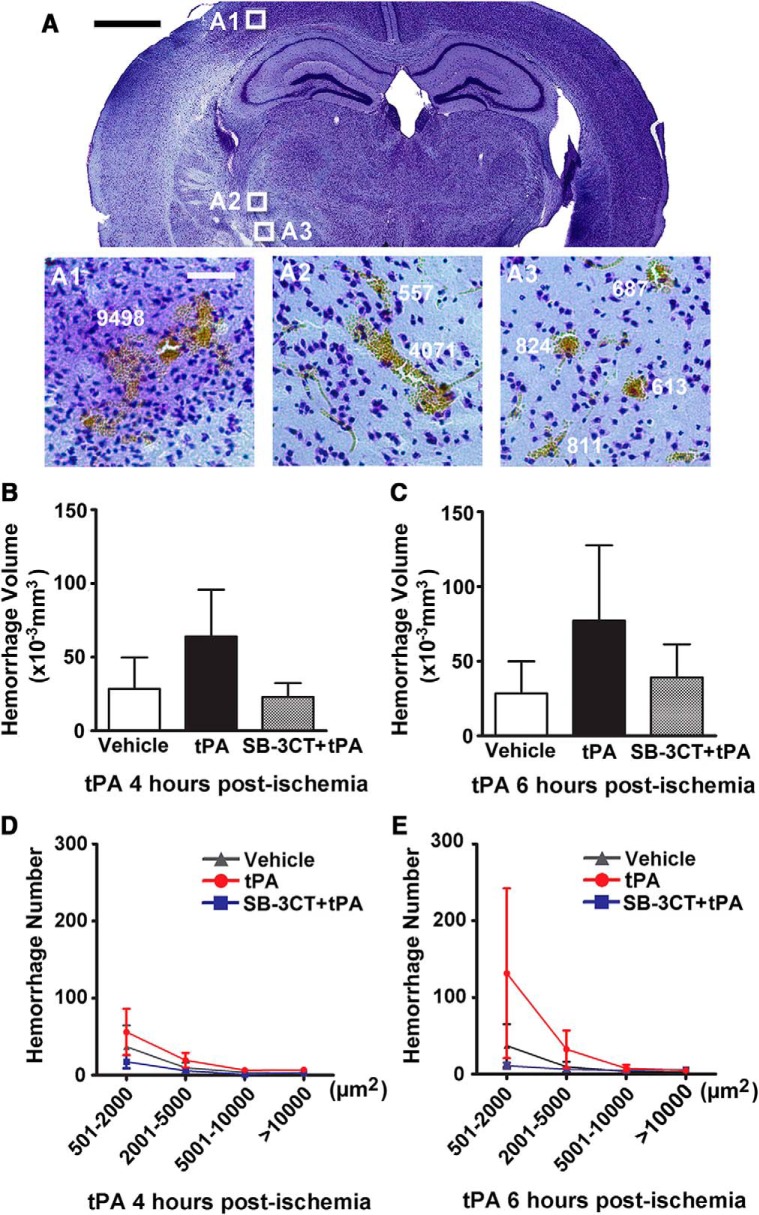
Effect of SB-3CT on ICH in tPA-treated mice after embolic ischemia. ***A***, Representative brain sections from the same mice described in Figure 1 revealed ICH of different sizes in the ischemic areas after embolic MCAO in mice. Scale bar: 1 mm. ***B***, ***C***, Quantification of hemorrhagic volumes. ICH was evaluated in cresyl violet-stained brain sections by bright-field microscopy in mice treated with saline or tPA at 4 h (***B***) or 6 h (***C***) postischemia. ICH volume was quantified using the stereology technique, with systematic sampling of 25–30 serial sections per brain. Each section was separated by 200-µm interval along the anteroposterial axis of the mouse brain; *n* = 10 mice in the vehicle-treated, *n* = 6 mice in the tPA-treated, and *n* = 7 mice in the SB-3CT + tPA-treated groups. ***D***, ***E***, Hemorrhage number of different areas in mice treated with tPA at 4 h (***D***) or 6 h (***E***) postischemia.

In this study, we also examined whether SB-3CT has any effect in reducing hemorrhage after delayed treatment with tPA by quantifying hemorrhage volume using the stereology technique on brain sections stained with cresyl violet, as described previously (Cui et al., 2012). Scattered hemorrhages of diverse sizes were identified and measured individually in the ischemic hemisphere (Fig. 3*A*). The tPA-treated mice showed larger total hemorrhage volume compared to the vehicle-treated mice, while the SB-3CT + tPA-treated mice showed smaller hemorrhage volume compared to mice given vehicle or treated with tPA at 4 h (Fig. 3*B*) and 6 h (Fig. 3*C*) postischemia. However, there were no significant differences due to large individual variations.

The spectrum of hemorrhagic transformation ranges from punctate or minor petechial bleeding (hemorrhagic infarct) to major mass-producing hemorrhage (parenchymal hematoma). Analysis of different sizes of hemorrhages was also performed to ascertain the incidence of ICH. Punctate or minor petechial hemorrhages ranging in size between 501 and 2000 µm^2^ were the most frequent (65.8% in mice treated with vehicle or with tPA at 4 h and 69.8% at 6 h postischemia). Additionally, more punctate or petechial hemorrhage was observed in the tPA-treated mice compared to the vehicle-treated mice, and less in the SB-3CT + tPA-treated mice (Fig. 3*D*,*E*). While no statistically significant differences were observed among the groups, there was a trend in increased ICH with delayed tPA treatment, as described in clinical trial studies (Hacke et al., 2008; Saver et al., 2013). In contrast, major mass-producing hemorrhage in the tPA-treated group did not show this pattern.

These data indicate that SB-3CT in combination with tPA protects against ICH after delayed administration of tPA, mainly by reducing punctate or petechial hemorrhage. This can be explained by hypoperfusion of the ischemic regions. Even with successful recanalization by tPA, distal vessels may not be reperfused sufficiently, resulting in persisting hypoperfusion due to the distorted microvasculature (An et al., 2011), such as pericyte contraction (Yemisci et al., 2009). SB-3CT is also demonstrated restoring the contraction of pericytes (Cui et al., 2012). Thus, the release of microcirculation from ischemia-induced neurovascular impairment may contribute to a decrease in punctate or petechial hemorrhage in the SB-3CT + tPA-treated mice.

### SB-3CT in combination with tPA attenuates MMP-9 expression in endothelial cells after embolic cerebral ischemia

**Figure 4.**
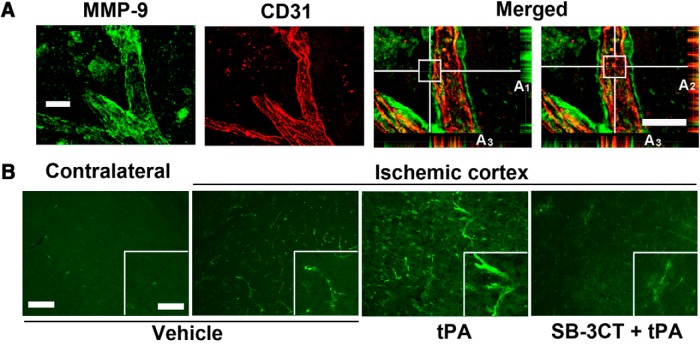
Effect of SB-3CT on tPA-induced MMP-9 expression in endothelial cells and vascular laminin after embolic ischemia. ***A***, Colocalization of MMP-9-positive immunoreactivity (green) with endothelial marker CD31 (red) in the ischemic penumbra. 3D deconvolution was used to enhance sharpness and contrast of fluorescent images. White horizontal and vertical lines indicate the sections of interest in the 3D structures of vessels. Radial and tangential planes, as well as the cross sections of the representative images, were achieved by orthogonal sectioning analysis and shown at the bottom right of the images: the tangential sections (***A_1_***), the radial sections (***A_2_***), and the cross sections (***A_3_***). ***B***, Photomicrographs of the ischemic penumbra and corresponding regions in the contralateral hemisphere for the different treatment groups. Scale bars: 20 μm (***A***), 100 μm (***B***), and 50 μm (inset).

Colocalization of MMP-9 immunoreactivity with the endothelial cell junction protein CD31 was observed, suggesting that endothelial cells are a significant source of MMP-9 after embolic cerebral ischemia (Fig. 4*A*). MMP-9 was predominantly observed at higher magnification in the tangential planes (Fig. 4*A1* ) and CD31 in the radial planes (Fig. 4*A2* ) of the vessels. Vessel cross-sectioning confirmed that the CD31 staining was sandwiched between two layers of MMP-9 (Fig. 4*A3* ). This study indicates that MMP-9 is secreted and localized at the ECM surrounding the endothelial cells after cerebral ischemia. Upregulation of MMP-9 expression in the mice treated with tPA at 6 h postischemia was observed, while SB-3CT in combination with tPA attenuated its expression (Fig. 4*B*).

### SB-3CT in combination with tPA protects against laminin degradation and endothelial cell loss after embolic cerebral ischemia

**Figure 5.**
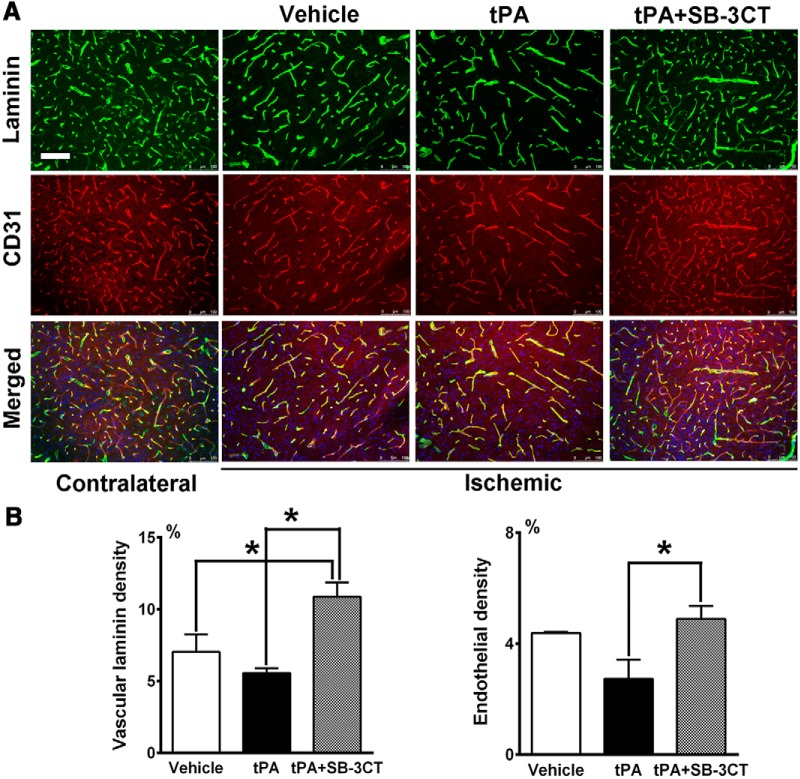
Effect of SB-3CT on tPA-induced degeneration of vascular laminin and endothelial cells after embolic ischemia. ***A***, Representative brain sections from the same mice described in Figure 4 were immunostained with anti-laminin antibody (green), endothelial cells with CD31 (red) and cell nuclei with Hoechst dye (blue). Scale bar: 100 μm. ***B,*** Density of laminin-positive vessels. ***C,***Density of CD31-positive vessels; *n* = 3 for vehicle, *n* = 6 for tPA, and *n* = 6 for SB-3CT + tPA groups; *p < 0.05 by one-tailed Student’s *t* test. Data are expressed as mean ± SEM.

The basal lamina is one of the major structures affected in tPA-induced BBB disruption (Yamashita et al., 2009). Laminin is a critical component of the basal lamina and serves as a cell-survival factor, preventing cells from degeneration (Frisch and Francis, 1994). Integrin-matrix interactions of the cerebral vasculature may explain how laminin degradation mediates the loss of endothelial cells after ischemia (del Zoppo and Milner, 2006). Therefore, we examined the effect of SB-3CT on laminin in mice treated with tPA at 6 h postischemia. After embolic cerebral ischemia, loss of vascular laminin as well as a decrease in CD31-positive endothelial cells were observed in the ischemic penumbra, compared to the corresponding contralateral regions. This reduction was more pronounced in the tPA-treated mice, compared to the vehicle group. SB-3CT in combination with tPA prevented laminin degradation and impairment of endothelial cells (Fig. 5*A*). Using ImageJ software with Vessel Analysis plugin, we measured the densities of laminin and CD31-positive vessels in multiple regions of the cortex penumbra. Combined treatment with SB-3CT + tPA significantly increased the density of laminin-positive vessels (10.9 ± 1.0%) compared to vehicle-treated mice (7.0 ± 1.2%, *p* < 0.05) and to tPA-treated animals (5.6 ± 0.3%, *p* < 0.001; Fig. 5*B*). Mice treated with SB-3CT + tPA showed prevention of the loss CD31-positive vessels (4.9 ± 0.5%) compared to mice treated only with tPA (2.7 ± 0.7%, *p* < 0.05; Fig. 5*C*). These data indicate that SB-3CT in combination with tPA protects against tPA-induced impairment of basal lamina and endothelial cells.

### SB-3CT in combination with tPA attenuates endothelial caveolin-1 expression after embolic cerebral ischemia

**Figure 6.**
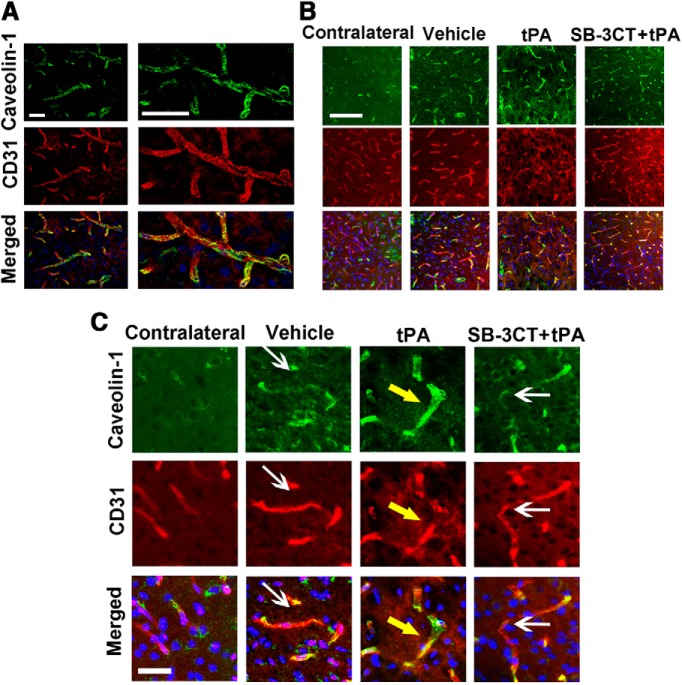
Effect of SB-3CT on tPA-induced increase of caveolae-mediated transcytosis after embolic ischemia. ***A***, Representative brain sections from the same mice described in Figure 4 were immunostained with caveolin-1 (green), CD31 (red), and Hoechst (blue) in the ischemic penumbra. 3D deconvolution was used to enhance the sharpness and contrast of fluorescent images. ***B***, Immunofluorescent staining showed increased caveolae-1 expression in the ischemic penumbra. Caveolin-1 expression became more pronounced with tPA treatment and decreased with SB-3CT + tPA treatment. ***C***, Magnified images from the different treatment groups. White arrows indicate areas of less caveolin-1 expression; yellow arrows indicate areas of enhanced caveolin-1 expression in the endothelial cells. Scale bars: 20 μm (***A***), 100 μm (***B***), and 25 μm (***C***).

Caveolin-1 plays an important role in early BBB breakdown, as it is a key mediator in the process of vesicular trafficking in the transcytosis of proteins (Nag et al., 2007; Knowland et al., 2014). Caveolae-mediated transcytosis in the nonlesioned region is relatively low, consistent with a previous report (Nag, 2003). Strong colocalization of caveolin-1 with CD31 immunoreactivity was observed by confocal microscopy, suggesting that caveolin-1 is mainly expressed in endothelial cells in the ischemic penumbra (Fig. 6*A*). Although there were less CD31-positive endothelial cells observed in the tPA-treated mice (Figs. 5, 6*B* , in red), the expression of caveolin-1 was considerably enhanced in the endothelial cells that survived (Fig. 6*B*, in green). SB-3CT in combination with tPA mitigated the upregulation of caveolin-1. Magnified images of the vessels revealed that caveolin-1 was not observed in the contralateral regions but was expressed along almost all of the length of the vessels in the tPA-treated mice, while it was partially expressed in the vehicle-treated or SB-3CT + tPA-treated mice (Fig. 6*C*). Increased caveolae-mediated transcytosis in the endothelial cells may serve as another mechanism of tPA-induced impairment to the neurovasculature.

## Discussion

tPA plays its therapeutic role by dissolving blood clots inside the blood vessel. However, tPA also induces potential neurovascular toxicity, if it leaks outside the blood vessel and reaches the parenchyma (Lo et al., 2004). In animal studies, tPA has been demonstrated to promote excitotoxin-induced neuronal degeneration and brain damage after cerebral ischemia (Tsirka et al., 1995; Wang et al., 1998). Delayed treatment with tPA may also be accompanied by degradation of ECM components and increased BBB permeability (Yepes et al., 2003; Su et al., 2008), as well as enhanced hemorrhagic transformation (Lapchak et al., 2000; Derex and Nighoghossian, 2008; Hacke et al., 2008).

In clinical trials, tPA administered between 3 and 4.5 h after the onset of stroke is more frequently associated with symptomatic ICH although improved overall clinical outcomes compared to placebo (Hacke et al., 2008). Even more severe ICH is observed if tPA is administered beyond 4.5 h. The undesirable symptomatic ICH of tPA is significantly related to the time of administration after the onset of ischemic stroke. Reduced mortality and fewer symptomatic ICHs are observed for every 15 min that tPA is administered earlier (Saver et al., 2013).

This temporal effect of tPA on the BBB integrity may be influenced by previous injury-induced alteration of the BBB (Benchenane et al., 2005). This is supported by an *in vivo* study that showed that increased BBB permeability was observed only when exogenous tPA was administered into the CSF, but not when it was given intravenously to nonischemic mice (Su et al., 2008). Also, in clinical practice, the risk of symptomatic ICHs after tPA treatment was found to be significantly higher in patients with more cerebral microbleeds present before administration of tPA (Tsivgoulis et al., 2016). Thus, it is reasonable to consider that the prerecanalization conditions of the cerebral microvasculature may affect the consequences of tPA treatment. In the early stage of ischemia, exogenous tPA mainly promotes its intravascular fibrinolysis function and has only limited capacity to impact the healthy BBB or the less-damaged BBB (Niego and Medcalf, 2014). Subsequent to the processing of cerebral ischemia, the following mechanisms are considered to contribute to the gelatinase-induced impairment of the neurovascular unit and increased BBB permeability: (1) progressive loss of basal lamina, a specialized layer of ECM, which leads to dysfunction of endothelial cells and loss of microvascular integrity (Hamann et al., 1995; del Zoppo et al., 1998) through disruption of integrin–matrix interactions in the ECM (Tagaya et al., 2001; Del Zoppo et al., 2006); (2) degradation of tight junction proteins such as occludin and ZO-1, unsealing the gaps between adjacent endothelial cells and increasing the paracellular permeability (Yang et al., 2007; Mishiro et al., 2012); (3) contraction and loss of pericytes (Dore-Duffy et al., 2000; Cui et al., 2012; Liu et al., 2012a) restrict CBF (Hall et al., 2014; Fernández-Klett and Priller, 2015), leading to persistent hypoperfusion in the ischemic area; and/or (4) enhancement of caveolae-mediated transcytosis of endothelial cells through disruption of pericyte-endothelial cells interaction (Brillault et al., 2002; Al Ahmad et al., 2009; Armulik et al., 2010; Daneman et al., 2010; Knowland et al., 2014).

**Figure 7.**
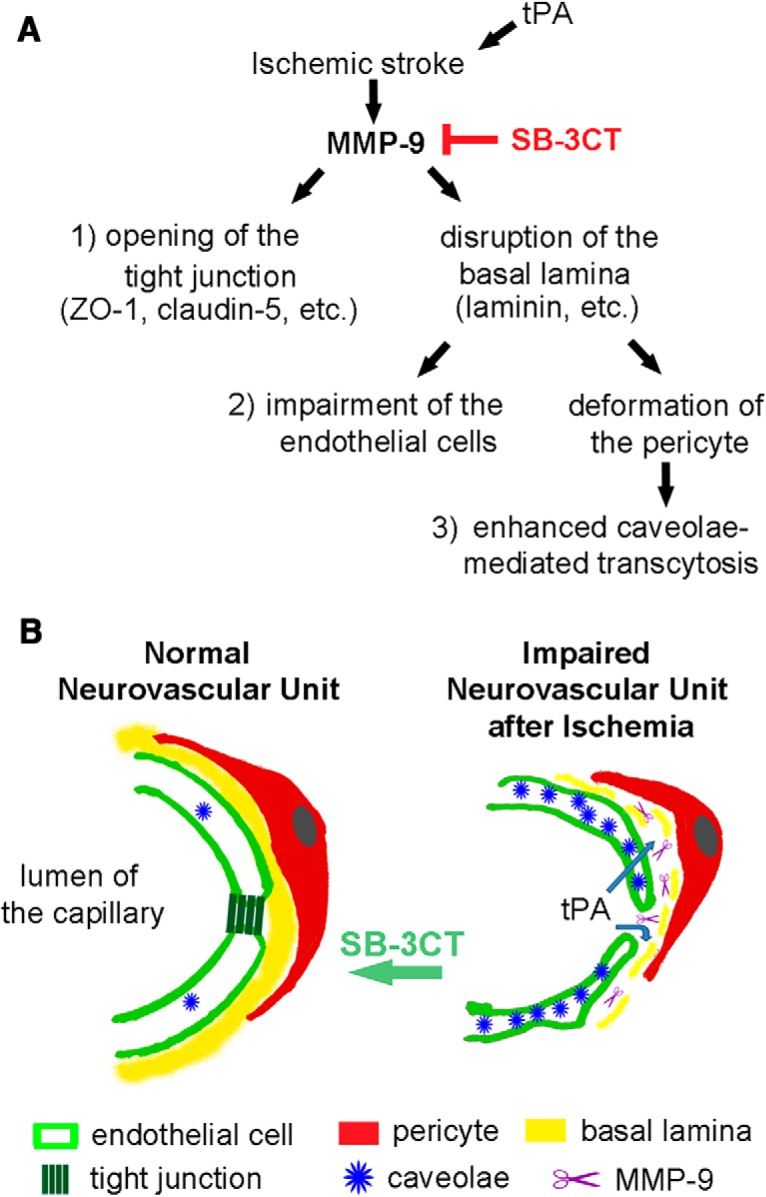
Model for MMP-9-mediated impairment of the neurovascular unit after ischemic stroke. ***A***, Schematic diagram shows impairment of the neurovascular unit due to upregulation of MMP-9 activity after ischemic stroke and exogenous tPA administration. ***B***, left, In the normal neurovascular unit, endothelial cells (light green) and pericytes (red) interact with a shared basal lamina (yellow). Tight junctions (dark green) seal the endothelial cells. Caveolae (blue) are expressed in low level. Right, In the impaired neurovascular unit after ischemic stroke, increased MMP-9 (purple) degrades the basal lamina, which in turn leads to the impairment of endothelial cells and deformation of pericytes. Detachment of the pericytes away from the endothelial cells contributes to the increase of caveolae. MMP-9 also breaks tight junctions. These mechanisms result in increased permeability of the BBB, followed by leakage of tPA to the abluminal side of the neurovascular unit and the brain parenchyma.

The stepwise molecular cascades in impairment of the neurovascular unit after ischemic stroke are illustrated in Figure 7*A*. In the normal neurovascular unit, endothelial cells and pericytes interact with the basal lamina, tight junctions seal the endothelial cells, and caveolae are expressed in low levels (Fig. 7*B*, left). Following ischemic stroke, the neurovascular unit is impaired, with increased MMP-9 activity degrading the basal lamina and tight junctions. This leads to impairment of endothelial cells and pericyte deformation, resulting in detachment of the pericytes from endothelial cells. Pericytes regulate the integrity of the BBB with an important inhibitory mechanism to reduce the number of caveolae and the rate of transcytosis (Al Ahmad et al., 2009; Armulik et al., 2010). Migration of pericytes away from the brain microvessels may relieve this inhibitory signaling pathway (Liu et al., 2012a). The dysfunction of the barriers in turn leads to the exposure of the abluminal side of the microvessels and the brain parenchyma to the components in blood plasma. Exogenous tPA could diffuse in the brain parenchyma and its deleterious effect could counteract its beneficial thrombolytic action (Fig. 7*B*, right). This hypothesis is supported by the current work, as well as previous studies suggesting that an exacerbation of tPA passage to the brain parenchyma through the injured BBB occurs only in the late stage of ischemia (Benchenane et al., 2005).

The exogenous tPA in the brain parenchyma amplifies MMP-9 levels and exaggerates the MMP-9-mediated impairment to the BBB consequently (Aoki et al., 2002; Tsuji K et al., 2005). Oxidative stress-mediated NF-κB transcription and low-density lipoprotein receptor-related protein may contribute to an increase of the tPA-induced gelatinases in the brain parenchyma (Wang et al., 2003; Yepes et al., 2003). This self-perpetuating loop of MMP-9 expression after tPA administration may explain why higher levels of MMP-9 expression predict more severe intracranial hemorrhage with thrombolytic treatment in human stroke (Montaner et al., 2003).

Therefore, early prevention of BBB disruption with MMP-9 inhibition before the tPA-mediated self-perpetuating loop of MMP-9 expression is of significant importance, because it may mitigate the bleeding-prone detrimental outcome among patients treated with intravenous tPA and extend the thrombolytic treatment time window. Administration of batimastat right after embolic ischemia in rabbits was found to decrease only the hemorrhage rate, but it did not reduce hemorrhage volumes nor infarct volumes (Lapchak et al., 2000). In the rat study, batimastat administered before embolic ischemia in combination with tPA significantly reduced hemorrhage volumes, but there was no effect on infarct volumes (Sumii and Lo, 2002). However, limitations of batimastat preclude it in clinical use. First, batimastat as a zinc chelator is a broad-spectrum MMP inhibitor with low specificity and thus potentially more side effects (Fingleton, 2008). Second, batimastat has a topological polar surface area of 108 Å^2^ [the upper limit for a molecule to penetrate the brain is 90 Å^2^ (Pajouhesh and Lenz, 2005), suggesting that it does not cross the BBB. The inability of >98% of small molecules to cross the BBB represents the greatest challenge in the development of neurotherapeutics (Pardridge, 2005).

SB-3CT has been shown to prevent proteolysis of the key constituent in basal lamina-laminin (Gu et al., 2005), to stabilize the tight-junctions (Liu et al., 2012b) and to restore pericyte contraction after cerebral ischemia (Cui et al., 2012). Moreover, SB-3CT has unique pharmacokinetic properties that make this compound privileged in the treatment of neurologic diseases. First, SB-3CT is the first gelatinase inhibitor with pathologically activated therapeutics (PAT) strategy. PAT is a novel neuroprotective strategy based on the principle that drugs should interact with their target only during states of pathologic activation but not interfere with the target if it functions normally. Such drugs should therefore exhibit little inhibition of normal physiologic function (Lipton, 2007). Pathologic activation of MMP-9 leads to opening of the SB-3CT thiirane-ring and tight-binding inhibition of the enzyme (Forbes et al., 2009). Moreover, SB-3CT is rapidly absorbed and avidly crosses the BBB, where it distributes in the brain at therapeutic concentrations (Gooyit et al., 2012). These favorable pharmacokinetic properties of SB-3CT resulted in efficacy in a mouse model of traumatic brain injury (Hadass et al., 2013). In this study, we demonstrated that SB-3CT in combination with tPA attenuated MMP-9-mediated degradation of endothelial laminin and impairment of endothelial cells, as well as decreased caveolae-mediated transcytosis. Early inhibition of MMP-9 activity by SB-3CT a selective mechanism-based inhibitor of gelatinases, decreased brain damage, reduced BBB disruption and prevented hemorrhagic transformation after delayed tPA treatment.

Thrombectomy in patients with disproportionately severe clinical deficit relative to infarct volume has been documented recently to result in better disability outcomes than those with standard of care alone, providing patients with a surgical option for treatment of ischemic stroke (Nogueira et al., 2018). We document that early administration of SB-3CT reduces the MMP-9 levels and mitigates the side effects associated with tPA. This combination treatment strategy holds great promise for both disease intervention and for benefiting a larger population of patients might qualify as recipients of the treatment.

## Synthesis

Reviewing Editor: Christophe Bernard, INSERM & Institut de Neurosciences des Systèmes Decisions are customarily a result of the Reviewing Editor and the peer reviewers coming together and discussing their recommendations until a consensus is reached. When revisions are invited, a fact-based synthesis statement explaining their decision and outlining what is needed to prepare a revision will be listed below. The following reviewer(s) agreed to reveal their identity: Gregory Bix.

The revised manuscript has addressed several of the major concerns that the reviewers had with the original submission. While one of the reviewers seems supportive of the publication of the manuscript, the second reviewer has raised several concerns regarding the scientific rigor of the study as follows:

Major:

1) The authors state on page 10 (line 214) that there was no significant difference in hemorrhage volume between the SB-3CT + tPA and the tPA alone groups (Fig 3 B and 3C) due to the large variation in the data. Were there any significant differences in Fig 3D and 3E? This is crucial as the concept of the study is the SB-3CT will be beneficial in reducing tPA-induced hemorrhage. Is the study under powered? How many animals were used in this study?

2) There are several problems with Fig 5. a) The description in the methods is inadequate. What is being measured in these data? I assume its area of staining/total area, but that should be stated.

b) I would have preferred vessel number or length/mm^2 (if vessel diameter is changing).

c) The figures should include the contralateral hemisphere.

d) Importantly, I am not sure about how to interpret the results. The authors propose that it reflects laminin degradation/ loss of CD31/ vessel loss. If there is laminin degradation/loss of CD31 the two appear to be going in tandem. I can't see any vessels that have staining for one and not the other. 24 hours seems quick for total vessel loss. An alternate explanation is that these changes might be related to edema and tissue swelling(vessel dispersion).

3) Another reviewer asked that the authors use an exogenous tracer to assess blood-brain barrier permeability rather than endogenous IgG. The authors tried that and in the response to the critique say that the experiment showed some animals with areas of very little leakage attributable to non perfusion of the ischemic tissue. Those results are important and should be reported. If vessels are damaged and non-perfused, they can not bleed and that may change with tPA-induced reperfusion. In addition, the exogenous tracer gives a measure of permeability.

Minor:

1) The Significance statement should be carefully read. The sentence from Line 22 to line 27 doesn't make sense to me as written.

2) The first sentence of the discussion could be better worded.
